# Supplementary material for: SeqScreen: accurate and sensitive functional screening of pathogenic sequences via ensemble learning
Source: Genome Biol. 2022 Jun 20;23:133. doi: 10.1186/s13059-022-02695-x (PMC9208262; doi:10.1186/s13059-022-02695-x)
Supplement: Supplementary file 2 — Additional file 2: Peer review history. [file 13059_2022_2695_MOESM2_ESM.docx]

**Review history**

**First round of review**

**Reviewer 1**

This is a well-designed and well-written study to address the gap for functional assignment of sequences of concern using a highly curated database of novel FunCoCs.

The methods, design, choice of and limitations of ML models were well addressed.

I recommend this manuscript for publication with only minor revisions of a few typos listed below and one clarification.

Can you please clarify input types for SeqScreen? It appears fasta is the primary input, although fastqs were used for the SARS-CoV-2 clinical example. Can individual sequence reads from fastqs be used as input as well? Can you please clarify in the paper?

Typos:
Page 4, Line 60 states you tested 11 ML models but Page 11, Line 1 states 10.
Page 5, Line 51, change "a hit the" to either "a hit to the" or just remove "a hit"
Page 16 Line 38, change "be" to "by"
Page 21, Line 52, change "fist" to "first"

**Reviewer 2**

The work by Balaji et al presents an upgraded version of SeqScreen for the identification of various pathogens from raw (short) sequencing data. The previous version of SeqScreen was already published on 2019 IEEE conference of BIBM. As a comparison, the major improvement of the current version is likely the development of FunSoCs dataset for pathogen detection. However, the manuscript provides very limited information about the construction of this key dataset (e.g., the original data sources and the enrollment criteria). The only valuable information is the brief descriptions of the 32 groups of FunSoCs in supplementary table 1. But the classification criteria of these groups are unclear as they cover viral, bacterial and eukaryotic pathogenesis and the table provides only one example for each. Since the current SeqScreen heavily depends on the customized FunSoCs dataset rather than any other published or widely used resources, the authors must justify the dataset in the manuscript or publish it elsewhere first. For example, why the P.aeruginosa aldolase PA3430 is classified into host cytoskeleton group? Please provide necessary reference(s).

The manuscript provides three cases to demonstrate the advantages of SeqScreen over previously published software. Unfortunately, none of them are convincible enough.
Case #1 is the raw sequencing data from the isolated and cultivated bacterial genomes. Such application is meaningless for pathogen detection in real world. Most current software are capable to identify the bacterial species correctly. The so called strain-level identification is usually unreliable. The author define sample Fig 6b as E.coli O157:H7 just based on the presence of stxB and espF. It is actually very speculative. Shiga toxin and LEE T3SS present in all enterohemorrhagic E. coli, which includes some other serotypes (e.g., O26, O103 and O111) rather than O157 only. Therefore, to determine it as O157 the author need to find the related genes for the specific O-antigen.
Case #2 is an unfair comparison. From table 3 both of the two previous software likely miss-identified C.botulinum as C.sporogenes. But it's because the author removed all sequences of C.botulinum from the database in order to simulate a novel pathogen. That means NO genomic information of C.botulinum available at all. So that's very reasonable to recognize it as the close relative species. However, the author actually did not provide the result of SeqScreen for this test by removing all information of C.botulinum from the FunSoCs dataset. From the results shown in Fig 6, SeqScreen distinguish C.botulinum and C.sporogenes mainly based on the presence/absence of botA, the known key virulence determinant of C.botulinum. Therefore, in case of novel pathogen (i.e., no botA information available) SeqScreen obviously cannot identify C.botulinum correctly. So, it can't show any advantage of SeqScreen as compared to previous software.
Case #3 is going to show application of SeqScreen for pathogen detection from clinical samples. But the example is inadequate since the initial detection of SARS-Cov-2 was already based on metagenomic approach last year (Nature 2020, 579:270). So, it cannot prove the advantage of SeqScreen over exists methods. Particularly, the author omitted other potential pathogens (e.g., bacteria or fungi) from the sample but focused on viruses only. It's not a real world scenario for unknown pathogen detection from clinical samples.

The authors claim SeqScreen as a tool specifically designed for both synthetic DNA and metagenomic functional screening. But neither explanation of the principle for synthetic DNA detection nor example to show the capability are provided. Similarly, the authors particularly include a group of antibiotic resistance genes in FunSoCs, but never discuss the potential of SeqScreen for the detection of drug resistance.

The performance of machine learning for FunSoC predictions is not well evaluated and likely been overestimated. As described in the text along with the data shown in table 1, the author didn't employ cross-validation as commonly used by ML studies. Thus, the performance shown in table 1 may biased due to the random dataset division. In addition, the authors never discuss the macro F1 score, which is an important indicator of ML models. From table 1, the macro F1 score of MV ensemble model is <0.8, even less than that of Bl. SVC+NN (OS) model. Indeed, the model is poorly performed in some (>5) groups probably due to the relatively small sample size as discussed in the text. The reviewer cannot evaluate it since no detailed information provided for these groups of FunSoCs (e.g., sample size).

The current software is very large in size (220 GB in disk space for dependencies and databases) and require many existing software. So it's almost impossible for this reviewer to test it locally. But from the FAQ page, the current version of SeqScreen likely can't process large datasets, such as millions of sequences from metagenomes or metatranscriptomes. It is contradict to the description in the manuscript for application in metagenomic sequencing data from clinical samples. Also, the manuscript never discuss about the runtime of SeqScreen. The speed of analysis is a critical feature for clinical use.

Several additional minor comments:
1, it is ambiguous to use the term "pathogenic sequences", the author never clearly define the range of pathogens SeqScreen is capable to identify throughout the manuscript. For example, besides viruses and bacteria, can SeqScreen recognize fungi and parasites?
2, the references are not correctly formatted.
3, figure 7 is not necessary.
4, supplementary table 2 and 3 are not fully visible.

**Authors’ response to reviewers**

# Reviewer #1:

This is a well-designed and well-written study to address the gap for functional assignment of sequences of concern using a highly curated database of novel FunCoCs.

The methods, design, choice of and limitations of ML models were well addressed.

I recommend this manuscript for publication with only minor revisions of a few typos listed below and one clarification.

**Q1**. Can you please clarify input types for SeqScreen? It appears fasta is the primary input, although fastqs were used for the SARS-CoV-2 clinical example. Can individual sequence reads from fastqs be used as input as well? Can you please clarify in the paper?

**R1**. We thank the reviewer for this important observation. SeqScreen takes only fasta files as inputs. In the SARS-CoV-2 clinical example, the fastq files were converted to fasta before submission to SeqScreen for processing. We’ve added a clarification into the methods section.

Typos:

Page 4, Line 60 states you tested 11 ML models but Page 11, Line 1 states 10.

We tested 11 models including the MV ensemble, we’ve changed the text to reflect this.

Page 5, Line 51, change "a hit the" to either "a hit to the" or just remove "a hit". Changed it to “a hit to the”.

Page 16 Line 38, change "be" to "by" Changed to “by”.

Page 21, Line 52, change "fist" to "first" Changed to “first”.

# Reviewer #2:

The work by Balaji et al presents an upgraded version of SeqScreen for the identification of various pathogens from raw (short) sequencing data. The previous version of SeqScreen was already published on 2019 IEEE conference of BIBM. As a comparison, the major improvement of the current version is likely the development of FunSoCs dataset for pathogen detection. However, the manuscript provides very limited information about the construction of this key dataset (e.g., the original data sources and the enrollment criteria). The only valuable information is the brief descriptions of the 32 groups of FunSoCs in supplementary table 1. But the classification criteria of these groups are unclear as they cover viral, bacterial and eukaryotic pathogenesis and the table provides only one example for each.

**Q1**. Since the current SeqScreen heavily depends on the customized FunSoCs dataset rather than any other published or widely used resources, the authors must justify the dataset in the manuscript or publish it elsewhere first. For example, why the P.aeruginosa aldolase PA3430 is classified into host cytoskeleton group? Please provide necessary reference(s).

**R1**. We thank the reviewer for the comment on the creation and contents of the FunSoC framework. In response, we have updated the examples in Table ST1 to be clearer and include references. We also encourage the reviewer to see our updated version of SD4, which should clarify how the UniProt queries were performed to populate the FunSoC training datasets (https://app.box.com/file/682048732910?s=3ahojrtpoqj5vcdbocd1tac1rrn5ocow). Since our submission of the manuscript, a mini review explaining high level concepts behind the FunSoC framework has also been published: https://journals.asm.org/doi/10.1128/IAI.00334-21. We have also included this citation in the updated manuscript. Further, we have also provided a link to the training data (Protein UniProt ID and FunSoC labels) as a link in the manuscript: https://rice.box.com/s/60380nrh1mqfib5yic8x4damycbje838. We hope that this list will help with the understanding of biocurator definitions for each of the 32 FunSoCs.

**Q2**. Case #1 is the raw sequencing data from the isolated and cultivated bacterial genomes. Such application is meaningless for pathogen detection in real world. Most current software are capable to identify the bacterial species correctly. The so called strain-level identification is usually unreliable. The author define sample Fig 6b as E.coli O157:H7 just based on the presence of stxB and espF. It is actually very speculative. Shiga toxin and LEE T3SS present in all enterohemorrhagic E. coli, which includes some other serotypes (e.g., O26, O103 and O111) rather than O157 only. Therefore, to determine it as O157 the author need to find the related genes for the specific O-antigen.

**R2**. We thank the reviewer for this important observation. The presence of Shiga toxin and LEE T3SS in other enterohemorrhagic *E.coli* is an extremely valid point. We would like to clarify the experiment a little more to help with its understanding. The motivation of SeqScreen in pathogen detection is underscored by the advantages of function-based identification of pathogenic markers over using canonical taxonomic approaches (and tools) rather than the identification or classification of particular pathogen species or strains. We have also added clarification in the results text **[Pg 5];**

The motivation of the following use-case underscored the advantages of FunSoC-based identification of pathogenic markers over using canonical taxonomic approaches. Note here that SeqScreen specifically reports on pathogenic functions of sequences and is not built for the task of bacterial species or strain-level typing. Thus, SeqScreen can help distinguish pathogenic sequences in near-neighbor microbes, which is beyond the capabilities of existing taxonomic classification methods.

Fig 6. describes the FunSoCs associated with Illumina sequences from eight bacterial isolate genomes. All had antibiotic resistance genes. In **Fig. 6** (a,b) we show a comparison of the commensal strain of *E. coli K-12 MG1655* versus the pathogenic strain *E. coli O157:H7.* Both strains reported the following four FunSoCs: *cytotoxicity*, *secreted effector, secretion,* and *antibiotic resistance*. SeqScreen reports the additional presence of *Shiga toxin subunit B* (*stxB*)[42] in pathogenic *E. coli O157:H7* that differentiates it from E*. coli K-12 MG1655*.

Taking the *E.coli* case as an example, as the reviewer correctly states, since species level calls are more accurate and strain-level identification is usually unreliable - most general taxonomic classification tools would either call both *E.coli* O157:H7 and *E.coli* MG1655 at species level (i.e., *E.coli*) or incorrectly classify the strain (Note: we present these results in Table. 2). In this case, the end-user does not get any information on pathogenicity, or gets the wrong information, as there is clearly a pathogenic strain of *E.coli* present. Our FunSoC framework on the other hand immediately identifies stxB and espF as pathogenic elements and this can alert the user about the presence of harmful genes usually associated with enterohemorrhagic strains of *E.coli*.

The broader question of mapping FunSoC hits to identify specific pathogens is interesting and is something we aim to incorporate in subsequent future work. The discussion section mentions this as a possible improvement to the software.

**Q3**. Case #2 is an unfair comparison. From table 3 both of the two previous software likely miss- identified C.botulinum as C.sporogenes. But it's because the author removed all sequences of C.botulinum from the database in order to simulate a novel pathogen. That means NO genomic information of C.botulinum available at all. So that's very reasonable to recognize it as the close relative species. However, the author actually did not provide the result of SeqScreen for this test by removing all information of C.botulinum from the FunSoCs dataset. From the results shown in Fig 6, SeqScreen distinguish C.botulinum and C.sporogenes mainly based on the presence/absence of botA, the known key virulence determinant of C.botulinum. Therefore, in case of novel pathogen (i.e., no botA information available) SeqScreen obviously cannot identify C.botulinum correctly. So, it can't show any advantage of SeqScreen as compared to previous software.

**R3**. We thank the reviewer for this note, as this gives us a chance to carry out an experiment to elucidate the strengths of SeqScreen as a tool for pathogen detection. We would also like to emphasize again that the use-case for SeqScreen is pathogen detection and not exact species or strain typing.

In response to this reviewer’s comment, we modified the DIAMOND database to remove all UniRef100 proteins belonging to *C.botulinum* and ran SeqScreen with this new database to see if SeqScreen would still capture botulinum neurotoxin-specific markers. As the results show in **Supplementary table ST6**, we can see that the FunSoC framework was successful in capturing presence of botulinum neurotoxin in *C.botulinum* and two associated FunSoCs (disable_organ and degrade_ecm) without requiring any specific sequences pertaining to *C.botulinum* in the database. The pairwise similarities between *BotA* and the hits (calculated using EMBOSS needle) to the modified database show that we can capture hits to around 50%-60% sequence similarity. We’ve also added text to discuss this **[Pg 7]:**

To further test SeqScreen’s ability to identify pathogenic markers in the absence of a particular taxa in the database, we focused on *C. botulinum,* which was the pathogen that proved most difficult for tools to place in the above experiments*.* We removed all UniRef100 proteins that had the taxid corresponding to *C. botulinum* from our database. We then ran SeqScreen on *C. botulinum* reads and analyzed FunSoCs associated with them, just as in Use Case #1. We found multiple hits to *BotA* homologs from other *Clostridium* species that were associated with the same FunSoCs as in the original experiment (i.e., C. *botulinum* present in the database) except for *virulence regulator* **(Supplementary Table ST6).** Interestingly, we even found a hit to the *TetX* toxin gene in the *disable organ* FunSoC associated with *Clostridium tetani*, which is a distant homolog of the *BotA* gene and even lies beyond its UniRef50 cluster. In contrast to the other tools, the FunSoC-based framework allows for accurate detection of pathogenic markers without needing the exact taxa to be present, particularly since sequences are more conserved in protein than nucleotide space.

One of the advantages of our FunSoC database is that we do not rely on a taxonomic database for identification of pathogenic markers. Since amino acids are more conserved than nucleotide sequences, we can map to other Botulinum neurotoxin homologs in the database and still detect pathogenicity in sequences.

**Q4**. Case #3 is going to show application of SeqScreen for pathogen detection from clinical samples.

(A) But the example is inadequate since the initial detection of SARS-Cov-2 was already based on metagenomic approach last year (Nature 2020, 579:270). So, it cannot prove the advantage of SeqScreen over exists methods.

**R4**. (A) We thank the reviewer for sharing an interesting prior work with us. However, we would like to emphasize a few points that might not have been conveyed in its entirety by the text.

First, the aim of this case study was to address the ability of SeqScreen to computationally detect pathogenic markers from clinical samples (in this case from COVID-19 patients) in the absence of the pathogen being present in the database (hence SeqScreen v1.2 was used whose database excludes SARS-CoV-2). Second, the paper shared by the reviewer first uses pan-CoV primers to run a targeted PCR to extract reads of interest to analyze and hence already had some prior knowledge of sequence of interest. This is in stark contrast to our analyses where we were able to identify, completely computationally, specific reads containing hits to specific proteins and associated GO terms and FunSoCs involved in SARS-CoV-2 pathogenicity from unbiased metatranscriptome sequencing. Further highlighting the sensitivity of SeqScreen, the SARS-CoV-2 reads were missed in the PBMC samples of the original Xiong *et al.* 2020 publication (i.e., relevant sentence from original publication = “Of note, the virus seems not to infect PBMC as that we failed to detect viral RNAs as well as ACE2 expression in PBMC”). Hence, SeqScreen offers a major advantage in clinical monitoring even in the absence of an a priori characterization of an emerging pathogen, especially in the early pandemic stages and is representative of a real scenario. We have modified the text to emphasize this point **[Pg 7, Pg 9]**.

**Supplementary Figure SF3.** Thus, in data from a large-scale unbiased metatranscriptome sequencing study, we were able to computationally identify sequences from an emerging pathogen that was not present in the SeqScreen database.

Further, from our experiment on metatranscriptomic PBMC samples[50], SeqScreen was able to identify reads from SARS-CoV-2 that were missed in original study where the authors did not find SARS-CoV-2 viral RNA in PBMC samples of COVID-19 patients. This underlines SeqScreen’s sensitivity and advantage over other approaches in clinical monitoring, even in the presence of an uncharacterized emerging pathogen.

(A) Particularly, the author omitted other potential pathogens (e.g., bacteria or fungi) from the sample but focused on viruses only. It's not a real world scenario for unknown pathogen detection from clinical samples.

(B) The SeqScreen analysis for this study, in fact, did contain analyses for bacteria, viruses and eukaryotes (including fungi). However, the data was subsequently filtered to focus on GO terms specific to viral reads in the cases, with the assumption that in this pandemic scenario the clinicians suspected a respiratory virus. We let the data speak for itself when we identified highly pathogenic proteins replicase polyprotein and spike protein with associated GO terms from bat coronaviruses, which were the most striking and interesting results that were subsequently validated by the alignments in **Figure 7** to the SARS-CoV-2 genome.

**Q5**. The authors claim SeqScreen as a tool specifically designed for both synthetic DNA and metagenomic functional screening. But neither explanation of the principle for synthetic DNA detection nor examples to show the capability are provided.

**R5**. We thank the reviewer for pointing out that we did not have a synthetic DNA screening use case, and we have since added a small case study to address this. We included four examples of SeqScreen-assigned threatening sequences (i.e., disable_organ FunSoC assigned) that would be missed by the current best practices in DNA biosecurity screening, and two examples of SeqScreen-assigned non-threatening sequences (i.e., no FunSoCs assigned) that the current guidelines would flag as dangerous. This illustrates differences in assigning threat by sequence function vs. taxonomic lists of Biological Select Agents and Toxin (BSAT) organisms.

As a proof of concept, we have also analyzed simulated reads from 16S rRNA genes taken from *B.anthracis*, *B.melitensis* and *B.pseudomallei* which are ubiquitously found in all bacteria and responsible for the initiation of protein synthesis. We simulated reads from these widely used marker genes used to identify bacteria given they are unlikely to have pathogenic functions; SeqScreen successfully assigned no FunSoCs to these sequences but had hits to the BSAT database. While this is admittedly a simple test, it highlights a very important point; ubiquitous sequences that are highly conserved between commensals and pathogens commonly trip up methods based on k-mer based taxonomic assignment and classification. We’ve added some text for this in the manuscript **[Pg 6]**

To simulate its utility for synthetic biology, we also tested SeqScreen on two datasets. The first contained four eukaryotic toxins from different organisms and two non- pathogenic proteins from the Vaccinia virus. We found that current BSAT-based screening pipelines overlooked the eukaryotic toxins and flagged the two viral sequences as dangerous. This is consistent with current best practice guidelines for screening synthetic sequences, but the current method focuses on flagging sequences that map to a short list of taxons of concern and lacks resolution to classify sequences at a functional level. SeqScreen was able to annotate each of the toxins with the *disable organ* FunSoC and had no FunSoCs associated with the two non-pathogenic sequences from the Vaccinia virus **(Supplementary Table ST4).** As a negative control, we also tested SeqScreen against 10K reads, simulated using *wgsim*[43], from 16S sequences of three BSAT agents *Bacillus anthracis, Brucella melitensis,* and *Burkholderia pseudomallei*. This resulted in several false positive calls to the BSAT database (mapped using Bowtie2), whereas SeqScreen had no false positives for any bacteria **(Supplementary Table ST5)**.

This new data can be found in the manuscript as **Supplementary Tables ST4 and ST5**.

**Q6**. Similarly, the authors particularly include a group of antibiotic resistance genes in FunSoCs, but never discuss the potential of SeqScreen for the detection of drug resistance.

**R6**. We thank the reviewer for the suggestion on including a discussion about antibiotic resistance. To include a small discussion about AR, we used the well known CARD database as a test dataset for SeqScreen to show its flexibility for classification of AR genes **(Supplementary Figure SF4)**. We first obtained the corresponding UniProt identifiers for entries in CARD. Out of a total of 4803 genes in CARD, we found that 2526 uniprot ids were not in our DB. Of the remaining 1245 were present in our DB and marked with the AR FunSoC while 312 were false negatives. In order to show the ability of SeqScreen to extend predictions to commonly used DBs we trained on 1247 CARD proteins already in our database (+ 963 negative samples) and tested it on 2083 CARD proteins (+ 311 negative samples). During training, 10-fold stratified CV was employed as the dataset was small. We observed that our prediction precision and recall on the withheld test set was 1.0 and 0.99 respectively compared to 1.0 and 0.12 before training, thus improving its ability to capture signatures from other curated DBs like CARD. We’ve added text for this in the manuscript **[Pg 8]**.

VFDB has been widely used but is limited by a lack of specificity regarding the inclusion and selection criteria for its sequences. PATRIC has some annotation of pathogenic sequences but is primarily focused on bacterial genomes. PHI-Base describes the result of pathogen-host interactions for pathogenic sequences following mutation. SeqScreen was designed to overcome some of these limitations through an iterative ensemble machine learning framework that leverages functional information combined with curations to identify FunSoCs. In addition to the databases discussed, we also analyzed the antibiotic resistance markers in the CARD database[55]. We found that several UniProt/UniParc identifiers (52%) from CARD were not present in our FunSoC database. We used this as a use case to elucidate the flexibility of re-training our ensemble model to improve coverage on a specific database. **Supplementary Figure SF4** shows that re- training our ensemble classifier on a small sample of CARD proteins can result in increased recall from 0.12 to 0.99 on annotating CARD with the *antibiotic resistance* FunSoC.

We do acknowledge that there are several recent tools that are more suitable for targeted antibiotic resistance detection analysis, including PARGT (Chowdhury, Call and Borschat 2020), DeepARG (Arango-Argoty et al. 2018), NanoARG (Arango-Argoty et al. 2019) and AMRFinderPlus (Feldgarden et al. 2021), but none of them address the question of identifying multiple different pathogenic signatures beyond AR. SeqScreen is meant to introduce a tool for sequence-level detection for a broader range of pathogenic markers associated with 32 different FunSoCs and does not solely focus on AR. This example both highlights our ability to build off of previous and ongoing efforts for pathogenic markers (such as ARGs) as well as provide a resource able to characterize functions of sequences of concern not captured by existing tools.

**Q7**. (A) The performance of machine learning for FunSoC predictions is not well evaluated and likely been overestimated. As described in the text along with the data shown in table 1, the author didn't employ cross-validation as commonly used by ML studies. Thus, the performance shown in table 1 may biased due to the random dataset division.

**R7**. We thank the reviewer for the suggestion to include using cross-validation (CV). CV is generally only used on training/validation sets to improve learning so the performance of our models on the completely withheld test set (Table 1) can only be a lower bound and not an overestimation. We didn’t employ cross-validation towards our final training pipeline due to a combination of design choices that we describe below:

- **Parameter tuning and training times:** Cross-validation is an effective method to carry out hyperparameter tuning; however, for final training it can result in extremely long training times and computation resources, especially in shared- clusters. Given the number of models we tested and size of our training matrix, this would significantly increase training times. To balance this, we only employed train-val-test splits for the final training pipeline to make it efficient and quick in case the end-user wants to re-train/modify the models.
- **Addressing variance through ensemble learning and choice of metrics:** While individual models could have small variances, a major benefit of our final ensemble model is that it serves as a bagging framework to reduce variance in individual models that may have occurred due to train-val-test splits. We have added this point into the text with relevant citations **[Citations 69, 70]**. In this way, ensemble model corrects for inherent variability that might be present in individual classifier results and reduces overestimation. Besides, given the wide range (8 different metrics) over which we evaluated the models, the models we chose were by far the best over other models (i.e TS NN had best precision, TS Bl. SVC had best recall and Bl.SVC + NN had a good balance across most FunSoCs).

The final metrics reported are on a completely withheld test dataset independent of train- val splits that are strictly restricted to the training space. Further, in contrast to popular train-val-test splits like 80%-10%-10% or 70%-15%-15%, we were careful to incorporate larger test (25%) and validation (18.25%) which is far greater than that obtained through individual k-fold splits.

(B) In addition, the authors never discuss the macro F1 score, which is an important indicator of ML models. From table 1, the macro F1 score of MV ensemble model is <0.8, even less than that of Bl. SVC+NN (OS) model.

We intentionally place more emphasis on PL Precision and Recall than macro metrics because we feel it is more informative of how we do on sequences known to have FunSoCs which is important for pathogen detection experiments. In fact, metrics computed at macro-level are more likely to be biased towards the majority class (in this case, samples containing no FunSoCs or not that particular FunSoC), which offers no information about how it does on sequences with pathogenic potential. We see that the balance of PL Precision and Recall are highest for the MV ensemble. Also, intuitively, in a pathogenic detection experiment, we place higher importance on reducing false negatives than false positives, as missing a pathogen is a higher risk than assigning a commensal organism with a FunSoC (the latter being reflected in the slightly lower macro metrics).

(C) Indeed, the model is poorly performed in some (>5) groups probably due to the relatively small sample size as discussed in the text. The reviewer cannot evaluate it since no detailed information provided for these groups of FunSoCs (e.g., sample size).

A wide number of factors could contribute to the performance of models on a particular FunSoC, including but not limited to, number of training samples, the uniqueness of GO terms and keywords belonging to the category, correlations/relationships between sets of GO terms etc. FunSoCs are manually curated labels, so we do expect to find variability in the performance of models across each of them. To address the question on the number of samples, we have added a table in **Supplementary Table ST8** that details the number of examples in our training dataset for each FunSoC.

**Q8**. The current software is very large in size (220 GB in disk space for dependencies and databases) and require many existing software. So it's almost impossible for this reviewer to test it locally. But from the FAQ page, the current version of SeqScreen likely can't process large datasets, such as millions of sequences from metagenomes or metatranscriptomes. It is contradict to the description in the manuscript for application in metagenomic sequencing data from clinical samples. Also, the manuscript never discuss about the runtime of SeqScreen. The speed of analysis is a critical feature for clinical use.

**R8**. We thank the reviewer for their interest in running our software, and we recognize that SeqScreen is computationally restrained by the tools and databases in its pipeline. These tools include widely adopted analysis tools like DIAMOND, which require significant memory for execution, and in this case an expansive protein database is required for operation with DIAMOND (default mode) or BLASTX (sensitive mode). While we recognize that the SeqScreen pipeline will not run on a laptop, the current pipeline will run on Linux servers that are standardly available to bioinformaticians. Also, the DB size can be reduced from 220GB to ~73GB if the user just wants to run default mode by removing the BLASTX DB.

To enhance usability of SeqScreen (along with all of its dependencies), we offer installations through conda (https://anaconda.org/bioconda/seqscreen), or it can be run as Docker or Singularity container if the image is pulled from the quay.io biocontainer site (https://quay.io/repository/biocontainers/seqscreen?tab=tags).

SeqScreen can process metagenomes or metatranscriptome data (as long as the input data is converted from fastq or other formats to fasta). We’ve added runtime and memory benchmarking for three different HMP samples (10K, 100K and 1M reads) to give a sense of compute resources **(Supplementary Figure SF5)**. On the FAQ page of the SeqScreen documentation, we have reworded our statement and clarified that it can take a long time to process large datasets (e.g., metagenomes) if users do not have access to an HPC or larger system to process their datasets. In the future, we plan to make SeqScreen more adaptable to faster processing of metagenomes.

Several additional minor comments:

1, it is ambiguous to use the term "pathogenic sequences", the author never clearly defines the range of pathogens SeqScreen is capable of identifying throughout the manuscript. For example, besides viruses and bacteria, can SeqScreen recognize fungi and parasites?

**Supplementary Table ST1** contains information on the source of representative proteins in each of the FunSoCs based on whether they are found in bacteria, viruses or eukaryotes. The majority of the FunSoCs are geared towards bacteria and viruses, but a significant number (18/31) also contain representation from eukaryotes (e.g., eukaryotic protein toxins). We have included some text in the manuscript where we reference Supplementary Table ST1 to help clarify this.

2, the references are not correctly formatted. Reference formatting has been fixed.

3, figure 7 is not necessary.

We thank the reviewer for this suggestion. We have moved the figure to the supplementary as **Supplementary Figure SF3**. We would like to emphasize that given our clarifications earlier, the figure is central to the validation of our findings. It is a novel finding, especially in the context of the original publication recognizing that the COVID-19 patient data failed to identify specific hits to SARS-CoV-2 in PBMC samples, which we were able to identify through SeqScreen and highlight in our study.

4, supplementary table 2 and 3 are not fully visible.

Thank you to the reviewer for pointing this out, and the formatting issue previously encountered while exporting to PDF has been fixed now.

**Second round of review**

**Reviewer 1**

-

**Reviewer 2**

The revised manuscript was largely improved and the authors replied most of my previous concerns. But after tried a few examples by this tool in the local system, I have some additional comments/suggests to the authors. I think the output of the SeqScreen need to be improved to efficiently filter and summarize the tedious results and provide only the valuable information for users, before it can be widely used in future applications.

The authors emphasized many times in the response that the SeqScreen was designed to provide function-based pathogenic markers rather than the identification of particular pathogen species or strains. It makes sense to me since the reliable conclusion of causative agent(s) within a clinical sample cannot just based on sequencing results, and the clinical phenotypes are always critical. After viewing the output HTML from SeqScreen, I noticed that it actually just provides some facts/evidences deduced from the sequences rather than makes any further conclusion about possible pathogen(s). I think that’s also the main reason why many other taxonomic-based tools miss-identified the true organisms in the cases described in the manuscript (they try to get a conclusion or candidates list), whereas the authors believe the SeqScreen was correct in all cases. In my opinion, the SeqScreen didn’t produce any final conclusion(s) about the possible pathogen(s) exist in each sample. That’s fine at least to me to leave the decision by the users themselves to incorporate additional clinical factors. However, I believe it’s necessary to clearly clarify this feature of SeqScreen in the manuscript as it is very different to most other canonical taxonomic tools.

Based on the aforementioned feature of SeqScreen the further biological interpretation of the output from this tool would require considerable background on microbial pathogenesis. Thus, it raised another concern about the users of this tool. The authors also admitted that the current software is computationally restrained by the tools and databases in its pipeline, so it’s not easy to be installed by common biologists. Actually, it took about one month of my postdoc (background in bioinformatics) to fully setup the SeqScreen pipeline in the local system. Therefore, in order to make this tool to be widely used in future I’d like to suggest the authors to consider setup a web-based server rather than a stand-alone software to ensure more microbiologists can try this tool in practical applications.

I have particular concerns on the output of this tool. I noticed the output report of SeqScreen actually provides all “valid” hits of each input sequences without any necessary summary. So it likely overlooks the importance of the quantity of valid hits on each individual gene for further interpretation. However, in many cases (particularly for metagenomic data), multiple independent reads are necessary to exclude potential false-positive due to sequencing errors or artificial sequences etc. In my opinion, that is the reason why in case #3, the original paper claimed they failed to detect viral RNA, whereas the authors believe the SeqScreen reported positive – there are actually only very few reads matched to coronaviruses in the sample. Indeed, in an attempt to suppress false-positive most canonical taxonomic tools may ignore these hits due to low quantity, while the SeqScreen likely doesn’t consider the quantity of hits at all, so it is capable to “find” SARS-Cov-2. However, it could be very speculative to make such conclusion just based on the two reads as shown in SF3 in many real world cases. In addition, such kind of reports with any hit of single read to be shown as “presence” may lead to many false-positive results (i.e. the co-existence of many different possible pathogens), causing difficulties in further biological interpretation. In the response to my previous question about case #3, the author claimed to focus on viruses only. But I believe the output on bacteria might be a disaster to the clinicians if using the same criteria to include any single read hits. Therefore, I believe the claimed “sensitivity” of this tools is actually a spin-off of high false-positive.

Surprisingly, I found NADH dehydrogenase gene from Pan troglodytes (chimpanzee) in my report. It’s obviously unrelated to the 32 FunSoCs and it’s very likely the common host contamination from metagenomic data. I believe such kinds of hits shouldn’t be included into the output by default. Since SeqScreen is a tool dedicate to the identification of pathogen sequences, if the tool cannot filter the raw hits efficiently and present only valuable information for the users, the biological interpretation of the tedious report would be impossible by microbiologists. So I think the output of SeqScreen need to be largely improved to make it a really useful tool for future application.

Some specific comments:
1, page 3 line 19, blastx searches are inherently slower than blastp. As the initialize step already produced 6-frame translations from the input sequences, why not just run blastp using these translated peptides to speed up the sensitive mode?

2, page 3 line 23, raw sequencing data is usually in FASTQ format with quality information, and many tools (e.g. Bowtie) can use the base quality information for better alignment. I highly recommend to allow user input in an optional FASTQ format.
The assumption that one protein-coding sequence is present within each query sequence is weird to me. Previous studies usually conduct initial assembly to produce large contigs for better follow-up analysis. This setting means user can only input the raw sequencing data (always in FASTQ format) rather than assembled contigs (usually in FASTA format). Why not use contigs as input?

3, page 3 line 59, the SeqMapper module is likely unrelated to FunSoC assignment, and the author also admit that the mapping to BSAT may include false-positive. Please justify the necessity of this module in the pipeline.

4, page 4 line 23, if the top 1% hits cover more different taxon, how will the pipeline assign the taxonomy label?

5, page 4 line 27, how does the so called “outlier detection” work? Likely no explanation.

6, page 4 line 33, it sounds likely that only the best hit will be reported? If so, why it likely uses different strategies for DIAMOND and blastn?

7, page 8 lines 44-46, it somehow confused me. If retraining the classifier on a small sample of CARD can improve the identification of AR sequences by the tool, why not just include them into your dataset?

8, page 8 lines 55-56, “SeqScreen provides an advantage in that it also reports the most likely strain-level assignments …” - I don’t think the tool provides any strain-level assignments.

9, page 9 lines 36-38, subsampling is unlikely an ideal solution for large dataset since the tool will report any single hits. For example, in case #3 only 2 reads match SARS-Cov-2 in the entire sample, if the user analyze only 1/10 of the data the coronavirus is very likely to be missed.

10, Fig4 shows that there are 93,283 samples in total for train/validation/test datasets. But I can’t find the origin of this number throughout the text.

11, some additional feedbacks/suggestions to the output HTML report:
a) by default, the main output is a very large table with >32 columns and up to 100 rows, but usually most cells are “0” without any valuable information. So it’s very inconvenient to navigate the scroll bars vertically and horizontally at the same time to find the sparse “1” cells in the report page. It would be nice to just ignore the input sequences with all 32 categories absent (i.e. “0”), and also automatically hide the columns (i.e. categories) absent from all sequences in this page view. Thus, the output table will be very concise and informative to provide users a better overview.
b) as an user, besides those numerous detailed information of each sequence I would like to also have a brief summary about my input data after analysis similar to the Fig. 6. That’s very important to help the users to produce possible conclusion. Otherwise, users will get lost in those details particularly for very large dataset. So, I suggest to provide an additional function or page to summarize the data.
c) I noticed there are many rows in my output show only input sequence ID with organism, gene and all FunSoC columns to be shown as “-“. Is there anything wrong or just means no significant hits for these sequences? If it’s the latter, why not just ignore them?
d) For unknown reason, the “Filter” cannot work in my system with the up-to-date version of Chrome (v99) – click it can popup the window but just do nothing to the main table after clicking the “Apply” button.

**Authors’ response to reviewers**

The revised manuscript was largely improved and the authors replied most of my previous concerns. But after tried a few examples by this tool in the local system, I have some additional comments/suggests to the authors. I think the output of the SeqScreen need to be improved to efficiently filter and summarize the tedious results and provide only the valuable information for users, before it can be widely used in future applications.

We thank the reviewer for the positive comments with respect to the revised manuscript. We have carefully reviewed all of the detailed comments and suggestions, and have responded and revised accordingly. In addition, we have took all of the comments with respect to the verbosity of SeqScreen output to heart and have implemented changes to the code which directly addresses this relevant concern. Finally, with respect to concerns to false positives being reported, we have included a detailed response (C3/R3) highlighting SeqScreen’s high recall and precision scores on individual sequences compared to state-of-the-art functional annotation and taxonomic classification tools.

**C1:** The authors emphasized many times in the response that the SeqScreen was designed to provide function-based pathogenic markers rather than the identification of particular pathogen species or strains. It makes sense to me since the reliable conclusion of causative agent(s) within a clinical sample cannot just based on sequencing results, and the clinical phenotypes are always critical. After viewing the output HTML from SeqScreen, I noticed that it actually just provides some facts/evidences deduced from the sequences rather than makes any further conclusion about possible pathogen(s). I think that’s also the main reason why many other taxonomic-based tools miss-identified the true organisms in the cases described in the manuscript (they try to get a conclusion or candidates list), whereas the authors believe the SeqScreen was correct in all cases. In my opinion, the SeqScreen didn’t produce any final conclusion(s) about the possible pathogen(s) exist in each sample. That’s fine at least to me to leave the decision by the users themselves to incorporate additional clinical factors. However, I believe it’s necessary to clearly clarify this feature of SeqScreen in the manuscript as it is very different to most other canonical taxonomic tools.

R1: We agree with the reviewer that this is an important distinction and have added the following text in the first paragraph of discussion. Our intention was not to be overly critical of existing taxonomic-based tools, in fact, by-in-large they do a good job at microbial genome classification at genus and species levels. However, we highlight limitations to emphasize the benefit of a FunSoC centric approach. Additional related discussion is added in response to C3.

“SeqScreen is different from using canonical taxonomic classifiers for pathogen detection in that it outputs functional information (best hit protein) and FunSoC information (pathogenicity) at read level instead of a candidate list of taxonomic labels.”

**C2:** Based on the aforementioned feature of SeqScreen the further biological interpretation of the output from this tool would require considerable background on microbial pathogenesis. Thus, it raised another concern about the users of this tool. The authors also admitted that the current software is computationally restrained by the tools and databases in its pipeline, so it’s not easy to be installed by common biologists. Actually, it took about one month of my postdoc (background in bioinformatics) to fully setup the SeqScreen pipeline in the local system. Therefore, in order to make this tool to be widely used in future I’d like to suggest the authors to consider setup a web-based server rather than a stand-alone software to ensure more microbiologists can try this tool in practical applications.

**R2: We thank the reviewer for bringing the issues with the installation to our attention. We agree with the reviewer that installation from source can required significant time. However, as described in the documentation (**[**https://gitlab.com/treangenlab/seqscreen/-/wikis/02.-SeqScreen-Dependencies**](https://gitlab.com/treangenlab/seqscreen/-/wikis/02.-SeqScreen-Dependencies)**,** [**https://gitlab.com/treangenlab/seqscreen/-/wikis/03.-Installation-and-Execution**](https://gitlab.com/treangenlab/seqscreen/-/wikis/03.-Installation-and-Execution) **) , we highly recommend that users install SeqScreen through bioconda to save time (takes only a few minutes when excluding the database) and ensure version compatibility of the various dependencies. As of 03/30/2022, SeqScreen has been downloaded and installed through bioconda over 15,000 times and thus represents a robust installation path for SeqScreen.**

To help further aid users with the installation process, in response to the reviewer feedback, we have also created a short installation walkthrough of SeqScreen installation,including running and visualization of the example test output here: <https://rice.box.com/s/mtzj1y4xvp6enl0kfpumijfyrahdu5am>.

And last but not least, we now host the entire SeqScreen DB on a fast cloud storage server (via Internet2) on Wasabi, which allows for downloads without egress. In our testing of the database download speeds, we recorded download times of less than 1 hour for the full database:

While we realize the download times will vary for each individual user, we were happy to now offer this to the SeqScreen users to help reduce download and installation time.

Summarizing, we strongly feel that the combination of (1) conda packaged installation process, (2) a new tutorial video on how to install SeqScreen combined with rich documentation on the wiki, in addition to (3) faster download times, will help facilitate the download and installation process.

**C3:** I have particular concerns on the output of this tool. I noticed the output report of SeqScreen actually provides all “valid” hits of each input sequences without any necessary summary. So it likely overlooks the importance of the quantity of valid hits on each individual gene for further interpretation. However, in many cases (particularly for metagenomic data), multiple independent reads are necessary to exclude potential false-positive due to sequencing errors or artificial sequences etc. In my opinion, that is the reason why in case #3, the original paper claimed they failed to detect viral RNA, whereas the authors believe the SeqScreen reported positive – there are actually only very few reads matched to coronaviruses in the sample. Indeed, in an attempt to suppress false-positive most canonical taxonomic tools may ignore these hits due to low quantity, while the SeqScreen likely doesn’t consider the quantity of hits at all, so it is capable to “find” SARS-Cov-2.

However, it could be very speculative to make such conclusion just based on the two reads as shown in SF3 in many real world cases. In addition, such kind of reports with any hit of single read to be shown as “presence” may lead to many false-positive results (i.e. the co-existence of many different possible pathogens), causing difficulties in further biological interpretation. In the response to my previous question about case #3, the author claimed to focus on viruses only. But I believe the output on bacteria might be a disaster to the clinicians if using the same criteria to include any single read hits. Therefore, I believe the claimed “sensitivity” of this tools is actually a spin-off of high false-positive.

R3: We thank the reviewer for their comments on the sensitivity vs specificity tradeoff while analyzing metagenomic data. We agree that most taxonomy-based tools could require the presence of a certain threshold of reads to call the presence of a particular pathogen. However, we reason below that SeqScreen manages this tradeoff well based on our experiments:

1. **We have tested SeqScreen extensively on popular benchmarking datasets for both functional and taxonomic classification. We chose the F1-score which is the harmonic mean of Precision (Specificity) and Recall (Sensitivity) as the metric to evaluate performance and tradeoff. Supplementary Figure SDF1 and SDF2 show that performance of SeqScreen has higher precision (lower false positives) than all state-of-the-art functional annotation tools on lengths equivalent to Illumina read lengths (100-200nt). Notably, these functional annotation tools included the top performers from the CAFA challenge. Furthermore, in Supplementary Figure SDF3 we see that across different metagenomic benchmarking datasets and compared to six different taxonomic classifiers SeqScreen’s performance matches or outperforms other classifiers on short read datasets.**

2. **Specifically in the case of the SARS-CoV-2 use case, we see that the best hit proteins had significant e-values 1.3e-09 and 5.8e-29 which are unlikely to be False Positives. We provide the pairwise alignment in the supplementary data for further support for trusting this local alignment. SeqScreen also allows users to define their own thresholds using the –evalue flag to filter results as needed.**
3. **As the reviewer suggested earlier, we leave the decision to the users themselves to incorporate any further clinical information to make appropriate decisions depending on the use case. SeqScreen fills an important gap in that it assigns functional annotations, pathogenic labels, at the read level and sensitively reports all possible information available from a read to the end users. We leave as future work automating clinically actionable reports from SeqScreen’s foundation, such as those that exist for a handful of specific pathogens on commercial platforms (such as Biofire).**

Taken together, and in response to the reviewer's concern, we have added the following text to the discussion:

“While SeqScreen is able to accurately and sensitively characterize individual reads, it does not generate clinically actionable reports with respect to pathogen presence/absence. We leave as future work leveraging the SeqScreen output into clinically actionable pathogen presence or absence calls, in tune with previous efforts in this area including laboratory validation and clinical samples [57].“

**C4:** Surprisingly, I found NADH dehydrogenase gene from Pan troglodytes (chimpanzee) in my report. It’s obviously unrelated to the 32 FunSoCs and it’s very likely the common host contamination from metagenomic data. I believe such kinds of hits shouldn’t be included into the output by default. Since SeqScreen is a tool dedicate to the identification of pathogen sequences, if the tool cannot filter the raw hits efficiently and present only valuable information for the users, the biological interpretation of the tedious report would be impossible by microbiologists. So I think the output of SeqScreen need to be largely improved to make it a really useful tool for future applications.

R4: We thank the reviewer for the suggestion to improve the output of SeqScreen. At present we intentionally retain both microbial and non-microbial classifications within SeqScreen as some users indicated an interest in using our tool for host-microbial interactions, where classification on both host and microbial sides are needed. However, we do agree with the reviewer that more filtering and formatting options will help the interpretability of results.

We have added an additional formatting script as part of the pipeline. Briefly, the script has added the following optional execution flags and parameters to SeqScreen:

1. **–format: [Value between 1-5]. Outputs report with specific formatting in addition to final report**
   a. **1: Original Format**
   b. **2: Reads with hits only**
   c. **3: Reads containing FunSoCs only**
   d. **4: Gene Centric output with number of reads containing the gene and e-value information**
   e. **5: Similar to 4 but only contains information of Genes with FunSoCs**
2. **–keep_taxon: [Comma separated taxids]. Keeps entries from only these taxids**
3. **–filter_taxon: [Comma separated taxids]. Filter entries from these taxids.**

An example of the number of records after formatting can be seen in the figure below. Specifically, for users mainly concerned with FunSoC containing genes, the output is now two orders of magnitude smaller (from 199,970 lines down to 1549).

We thank the reviewer for this feedback, and feel that these additional parameters and options that we implemented in response to the feedback will enable improved analysis of SeqScreen output for downstream applications, including the ability to filter taxa that likely represent contamination/background.

Some specific comments:

**C5:** 1, page 3 line 19, blastx searches are inherently slower than blastp. As the initialize step already produced 6-frame translations from the input sequences, why not just run blastp using these translated peptides to speed up the sensitive mode?

R5: We thank the reviewers for their observation on blastx and six frame translations used in our pipeline. We would like to note here that since the frame is unknown we would have to run blastp on all six frames to get the appropriate result which is essentially the same as running blastx on the sequence. If the frame of translation was known beforehand, using an ORF caller for instance, then we fully agree with the reviewer that blastp would be a more viable alternative.

**C6:** 2, page 3 line 23, raw sequencing data is usually in FASTQ format with quality information, and many tools (e.g. Bowtie) can use the base quality information for better alignment. I highly recommend to allow user input in an optional FASTQ format.

R6: We thank the reviewer for the suggestion to support other input types to the software. Currently, we decided to keep the FASTA format because while DIAMOND/ Bowtie2 accepts FASTQ, BLAST used in SeqScreen sensitive mode only accepts FASTA, hence to keep information consistent between modes we used an uniform input (FASTA) format. Users may want to preprocess their FASTQ files based on quality using tools like Trimmomatic (used in the experiments in the manuscript) and convert their FASTQ to FASTA using highly popular command line tools for ([seqtk](https://anaconda.org/bioconda/seqtk), [seqkit](https://anaconda.org/bioconda/seqkit)) before analysis with SeqScreen. Thanks to the reviewer’s request, we do plan to include an option for FASTQ in our future release for SeqScreen fast mode and have opened a feature request on our [issues page](https://gitlab.com/treangenlab/seqscreen/-/issues/149) (<https://gitlab.com/treangenlab/seqscreen/-/issues/149>) for the same.

**C7:** The assumption that one protein-coding sequence is present within each query sequence is weird to me. Previous studies usually conduct initial assembly to produce large contigs for better follow-up analysis. This setting

means user can only input the raw sequencing data (always in FASTQ format) rather than assembled contigs (usually in FASTA format). Why not use contigs as input?

R7: We thank the reviewer for the suggestion of including contigs as input. This is fundamentally different from the current use-case that SeqScreen was designed for, summarized as follows:

1. **SeqScreen was designed as a short sequence classifier at the gene level. This stems from the motivation of creating SeqScreen; screening individual protein-coding DNA sequences for improved biosecurity (beyond the current status quo of BSAT based screening). This original use case naturally extends out to short-reads (obtained from Illumina etc.), and then it is assumed that one gene (or a part of it) is present within each query sequence. For any sequence that is not protein coding, we also include centrifuge as part of the SeqScreen framework to provide taxonomic classification even when no protein coding sequence is identified.**
2. **Contigs, in contrast to short-reads, could contain multiple genes within a given sequence which requires a specialized algorithm for taxonomic and functional analysis as seen in von Meijenfeldt et al. (2019) and Mirdita et al. (2021).**
3. **Contig output varies with the choice of the assembler which might influence the results on the same dataset. Also, making assembly part of this pipeline would be non-optimal because of the computational overhead needed and possibility for introducing assembly error (especially for low coverage datasets, metagenomic datasets, or disjoint reads).**

For these reasons, we leave extending SeqScreen’s capabilities on contigs as future work. Note, in response to this comment, we’ve already started laying the foundation via a few commits to the code in the latest versions (>v2.0) that we intend to test soon, which will allow us to expand SeqScreen to include contigs as input.

**C8:** 3, page 3 line 59, the SeqMapper module is likely unrelated to FunSoC assignment, and the author also admit that the mapping to BSAT may include false-positive. Please justify the necessity of this module in the pipeline.

R8: SeqMapper is a module that maps sequences to the BSAT database. This database is an industry standard for triaging sequences of concern. Furthermore, mapping reads to reference genomes is common to several microbial genome analysis tools. We include this as a baseline screening tool

as well as for the users to look at SeqScreen results alongside the simplistic SeqMapper results, we have enabled that option if they run sensitive mode, else its disabled.

We’ve added the following text under the SeqScreen workflow #2: SeqMapper

“Since the BSAT database is the industry standard for triaging sequences of concern, we use this as a baseline screening tool for the users to compare SeqScreen results alongside the simplistic SeqMapper results.”

**C9:** 4, page 4 line 23, if the top 1% hits cover more different taxon, how will the pipeline assign the taxonomy label?

R9: We thank the reviewer for an opportunity to clarify the taxonomic assignments made by SeqScreen. Currently, as part of our final output we have the *multi-taxids_confidence* column that indicates the taxonomy label of a sequence. If several taxonomy ids were found to be appropriate, then all of them with their corresponding confidence values are listed under the column. There is also an intermediate *taxonomic_results.txt* file that contains taxonomy labels from Centrifuge and DIAMOND separately (in fast mode) and from BLASTX (in sensitive mode) that the user can access.

**C10:** 5, page 4 line 27, how does the so called “outlier detection” work? Likely no explanation.

R10: We thank the reviewer for highlighting the “outlier detection” step. The publication linked to the code is already included as Reference [31]. We’ve also added some information about the outlier detection step in the text:

“The outlier detection uses a multiple-alignment scoring function, modified Bayesian Integral Log-Odds, to define the outliers within a subset of top BLAST (https://github.com/marbl/ATLAS) and further details can be found in Shah et al. (2018) [31].”

**C11:** 6, page 4 line 33, it sounds likely that only the best hit will be reported? If so, why it likely uses different strategies for DIAMOND and blastn?

R11: We thank the reviewer for the comment on BLASTN. In the sensitive mode, BLASTN is not used for best hit protein selection; rather we use BLASTX (Sensitive) and DIAMOND (Default), hence it has different strategies for classification. BLASTN + Outlier detection is used primarily for taxonomic classification of non-protein sequences. We have clarified this in the text:

“The *sensitive* mode (using the *--sensitive* flag) uses much more accurate and sensitive BLASTN-based alignments[30] and outlier detection[31] steps for taxonomic characterization in addition to BLASTX which is used to identify best hit proteins.”

In addition, we have made BLASTN optional (--blastn flag) starting with version v1.5.9.

**C12:** 7, page 8 lines 44-46, it somehow confused me. If retraining the classifier on a small sample of CARD can improve the identification of AR sequences by the tool, why not just include them into your dataset?

R12: We thank the reviewer for this suggestion. We mention in the manuscript that a majority of the CARD proteins (52%) are not in our database currently, since the SeqScreen database is limited to well annotated proteins with annotation score 3 and above. We are however in the process of expanding our database for future releases (v3.0 and above) and will look into updating our database with CARD annotations if they have sufficient annotation quality.

**C13**: 8, page 8 lines 55-56, “SeqScreen provides an advantage in that it also reports the most likely strain-level assignments …” - I don’t think the tool provides any strain-level assignments.

R13: The reviewer is absolutely correct in that SeqScreen does not always report strain level assignments at the read level, as we mentioned elsewhere in the text. We fixed this to now read:

“..in that it also reports the most likely taxonomic assignments …”

**C14:** 9, page 9 lines 36-38, subsampling is unlikely an ideal solution for large dataset since the tool will report any single hits. For example, in case #3 only 2 reads match SARS-Cov-2 in the entire sample, if the user analyze only 1/10 of the data the coronavirus is very likely to be missed.

We thank the reviewer for the important observation on subsampling as a solution for larger datasets. In contrast to random subsampling, the method cited, RACE, uses a diversity sampling method which only discards reads that are similar to the ones already found in the sample thereby having the ability to downsample while retaining sequence level diversity. We’ve added some clarification towards this in the text:

“To address this, one possible solution is to use a read or database subsampling method such as RACE[56]. In contrast to random sampling methods, methods like RACE employ diversity sampling using techniques like Locality Sensitive Hashing (LSH) to only discard reads that are similar to ones already present in the sample. Thus, this strategy shows potential to preserve the full complement of taxonomic and functional diversity while drastically reducing runtime.”

**C15:** 10, Fig4 shows that there are 93,283 samples in total for train/validation/test datasets. But I can’t find the origin of this number throughout the text.

R15: We thank the reviewer for pointing this out. We’ve added information about the dimensions of the matrix size in the Discussion as given below:

“The feature matrix had a size of 98,283 samples and 16,071 after pre-processing. This was the final matrix for downstream Machine Learning tasks.”

**C16:** 11, some additional feedbacks/suggestions to the output HTML report:

a) by default, the main output is a very large table with >32 columns and up to 100 rows, but usually most cells are “0” without any valuable information. So it’s very inconvenient to navigate the scroll bars vertically and horizontally at the same time to find the sparse “1” cells in the report page. It would be nice to just ignore the input sequences with all 32 categories absent (i.e. “0”), and also automatically hide the columns (i.e. categories) absent from all sequences in this page view. Thus, the output table will be very concise and informative to provide users a better overview.
b) as an user, besides those numerous detailed information of each sequence I would like to also have a brief summary about my input data after analysis similar to the Fig. 6. That’s very important to help the users to produce possible conclusion. Otherwise, users will get lost in those details particularly for very large dataset. So, I suggest to provide an additional function or page to summarize the data.
c) I noticed there are many rows in my output show only input sequence ID with organism, gene and all FunSoC columns to be shown as “-“. Is there anything wrong or just means no significant hits for these sequences? If it’s the latter, why not just ignore them?
d) For unknown reason, the “Filter” cannot work in my system with the up-to-date version of Chrome (v99) – click it can popup the window but just do nothing to the main table after clicking the “Apply” button.

R16: We thank the reviewer for their valuable suggestions to improve the SeqScreen output. We are always looking to improve visualization of the HTML output. We are happy to report that parts (a), (b) and (c) have been addressed with regards to the SeqScreen output (TSV) file. We will be looking into adding support of these formats to the HTML in our next release. We are sorry that the reviewer experienced problems with the filter option. As seen in the video demo, the filter option worked fine with Chrome v99, so we were unfortunately unable to reproduce the observed error and debug without further information. Note, we have occasionally seen the HTML page become unresponsive for extremely large outputs, which could be causing the issue the reviewer reported. We are currently working on a fix to address this issue to avoid this from occurring in the future.
